# Retraction: Awareness and infection prevention practices of hepatitis B virus among informal caregivers in public hospitals of Addis Ababa, Ethiopia, 2024

**DOI:** 10.3389/fepid.2026.1798141

**Published:** 2026-02-02

**Authors:** Frontiers Editorial Office

Following publication, concerns were brought to our attention regarding the authors' use and publication of primary research data without permission and without a mention to their contributors. Unauthorized data use is a breach of Frontiers' guidelines and those of the Committee on Publication Ethics. The authors have not provided a satisfactory response to confirm legitimacy of the study, as such, this article is being retracted.

In addition, this study was simultaneously submitted in BMC Infectious Diseases [Kasse, T., Solomon, T., Mesfin, A. et al. Awareness and infection prevention practices of hepatitis B virus among informal caregivers in public hospitals of Addis Ababa, Ethiopia, 2024: a cross-sectional study. BMC Infect Dis 25, 57 (2025). https://doi.org/10.1186/s12879-025-10477-6], which has since been retracted for duplicate publication.

This retraction was approved by the Chief Executive Editor of Frontiers. The authors have not responded regarding this retraction. Frontiers would like to thank Habtamu Sewunet for contacting the journal regarding the published article.

